# Suppression of deep-level traps via semicarbazide hydrochloride additives for high-performance tin-based perovskite solar cells

**DOI:** 10.1007/s12200-023-00103-1

**Published:** 2023-12-29

**Authors:** Wenbo Jia, Yi Jing, Han Zhang, Baoyan Tian, Huabo Huang, Changlei Wang, Ligang Xu

**Affiliations:** 1 Key Laboratory for Organic Electronics and Information Displays (KLOEID) and Jiangsu Key Laboratory for Biosensors, Institute of Advanced Materials (IAM), Nanjing University of Posts and Telecommunications, Nanjing, 210023 China; 2 Hubei Key Laboratory of Plasma Chemistry and Advanced Materials, Key Laboratory for Green Chemical Process of Ministry of Education, School of Materials Science and Engineering, Wuhan Institute of Technology, Wuhan, 430205 China; 3 School of Optoelectronic Science and Engineering and Collaborative Innovation Center of Suzhou Nano Science and Technology, Key Lab of Advanced Optical Manufacturing Technologies of Jiangsu Province and Key Lab of Modern Optical Technologies of Education Ministry of China, Soochow University, Suzhou, 215006 China; 4 Wuhan National Laboratory for Optoelectronics, Huazhong University of Science and Technology, Wuhan, 430074 China

**Keywords:** Lead-free perovskite solar cells, Deep-level traps, Power conversion efficiency, Semicarbazide hydrochloride, Stability

## Abstract

**Abstract:**

Tin perovskites with exemplary optoelectronic properties offer potential application in lead-free perovskite solar cells. However, Sn vacancies and undercoordinated Sn ions on the tin perovskite surfaces can create deep-level traps, leading to non-radiative recombination and absorption of nucleophilic O_2_ molecules, impeding further device efficiency and stability. Here, in this study, a new additive of semicarbazide hydrochloride (SEM-HCl) with a N–C=O functional group was introduced into the perovskite precursor to fabricate high-quality films with a low concentration of deep-level trap densities. This, in turn, serves to prevent undesirable interaction between photogenerated carriers and adsorbed oxygen molecules in the device’s operational environment, ultimately reducing the proliferation of superoxide entities. As the result, the SEM-HCl-derived devices show a peak efficiency of 10.9% with improved device stability. These unencapsulated devices maintain almost 100% of their initial efficiencies after working for 100 h under continuous AM1.5 illumination conditions.

**Graphical Abstract:**

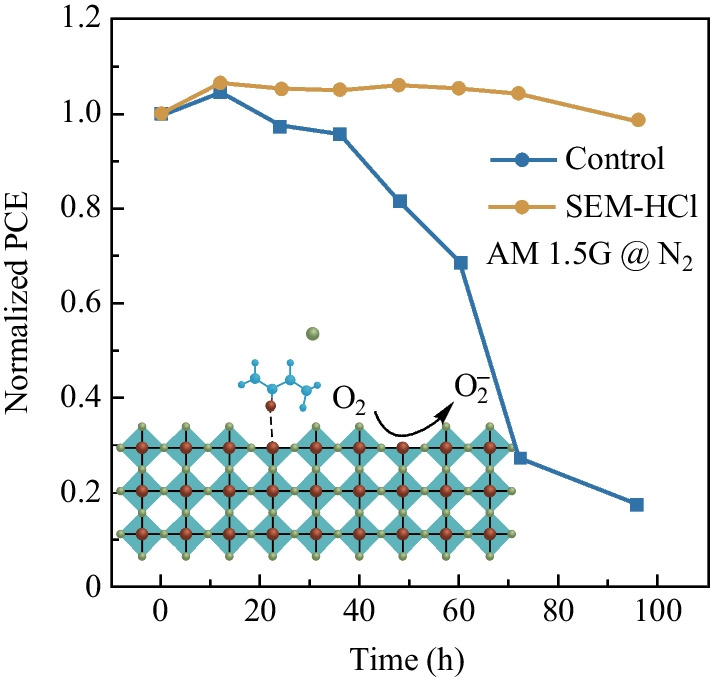

**Supplementary Information:**

The online version contains supplementary material available at 10.1007/s12200-023-00103-1.

## Introduction

Tin-based perovskite solar cells (TPSCs) have developed rapidly in the past five years generating power conversion efficiencies (PCEs) approaching 15% [1–7]. However, the potential of TPSCs remains constrained by their comparatively lower power conversion efficiency (PCE) when contrasted with lead-based PSCs, which frequently attain PCE values surpassing 25% [8–13]. This limitation is attributed to the incorporation of Sn ions in perovskite structures. It arises from two main mechanisms: (1) a low defect tolerance leading to the formation of high-density Sn vacancies [14–16]; (2) the easy oxidation of Sn^2+^ ions, resulting in self-doping effects [17–19]. Given these origins, it becomes inevitable to cultivate defects on both the surface and within the interior of the perovskite crystal grains, wherein the internal Sn vacancy defects tend to generate *p*-type doping characteristics [20–24].

Previous investigations have shown that providing an Sn-rich environment (by means of tin halides additives) is an effective strategy for inhibiting the formation of internal vacancies [22, 25–30]. In contrast to interior defects, Sn vacancies and undercoordinated Sn ions located on the perovskite surfaces can create deep-level traps, serving as prominent non-radiative recombination centers [31]. As a result, the defects of Sn vacancies and undercoordinated Sn ions have a significant influence on both the open-circuit voltage (*V*_oc_) and the PCE [32–34]. Furthermore, the unsaturated Sn dangling bonds on the perovskite surfaces are susceptible to the adsorption of various nucleophilic molecules (such as oxygen), and this process readily forms dative bonds with the Sn perovskite. Previous studies have demonstrated that, upon photoexcitation of perovskites, the surface-localized excitons or carriers migrate to the adsorbed oxygen molecules, converting them into superoxide (O_2_^−^). This transformation subsequently induces Sn^2+^ oxidation and the formation of deep-level traps [35, 36]. From an energetics perspective, the presence of superoxide molecules is significantly more conducive than that of oxygen molecules to the degradation of tin perovskite films. Meanwhile, the dimensional compatibility between superoxide molecules and iodide ions makes the former better suited for coordinating with unbound Sn^2+^, thereby facilitating the formation of an octahedral configuration. Consequently, the employment of efficient technologies targeted at reduced deep-trap states of the surface is considered essential for enhancing device performance and prolonging the operational lifespan of TPSCs [37]. But there is currently a lack of optimization strategies addressing the interaction between surface carriers and adsorbed oxygen molecules. Consequently, the development of an effective amelioration approach is urgently needed.

In this study, we employ a new additive, semicarbazide hydrochloride (SEM-HCl) with a N–C=O functional group, to fabricate high-quality tin perovskite films with low deep-level trap state densities. Opting for additive engineering rather than surface engineering offers a dual advantage. First, due to the insolubility of SEM-HCl in prevalent antisolvents like chlorobenzene (CB), this approach is more economically viable. Secondly, additive engineering transcends mere surface interactions; not only does it diminish the quantity of uncoordinated Sn^2+^ on the periphery, but it also modulates the intrinsic Sn deep-level defects. Such comprehensive modulation culminates in enhanced device performance. Moreover, SEM-HCl with O=C–N functional group can form coordination interactions with charge defects of the tin perovskites, thereby intensifying the electron cloud density surrounding the defects and enlarging vacancy formation energies. Crucially, this approach aids in the reduction of the deep-level trap state density, originating from undercoordinated Sn^2+^ ions and Sn^4+^ oxidation, which effectively reduces nonradiative recombination and extends the charge lifetime. As the result, the inverted TPSCs based on SEM-HCl additive deliver a PCE approaching 11%, accompanied by an extended lifespan approaching that of the initial PCE after 100 h under the continuous illumination.

## Results and discussion

In this study, SEM-HCl was introduced into tin perovskite precursor to reduce deep-trap states of the surface. And the tin perovskite films were fabricated via a one-step spin coating method using FA_0.75_MA_0.25_SnI_3_ in a mixture solution with 3 mol% of SEM-HCl additive. The tin perovskite wet films were treated by chlorobenzene antisolvent followed by 70°C annealing to enhance the perovskite crystallization.

**Fig. 1:**
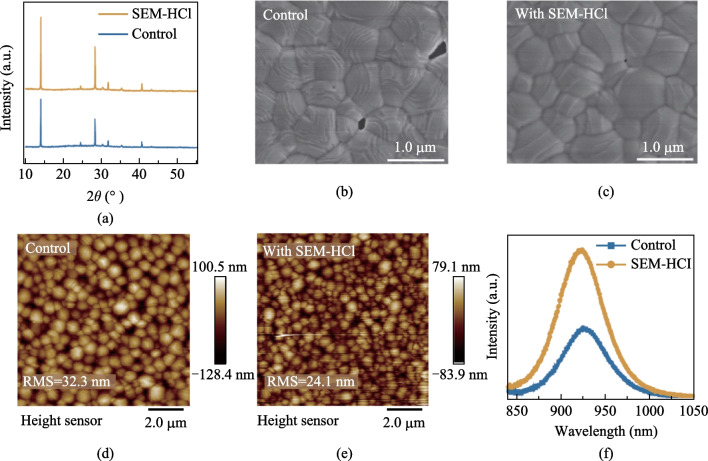
**a** XRD patterns. **b, c** Top-view SEM images. **d, e** AFM images and **f** steady-state PL spectra of the control and SEM-HCl-derived perovskite films

To illustrate the effects of SEM-HCl additive on the tin perovskite crystallization, X-ray diffraction (XRD) patterns of both the control and SEM-HCl-treated FA_0.75_MA_0.25_SnI_3_ are shown in Fig. 1a. The SEM-HCl-derived perovskite sample manifested a typical XRD pattern analogous to that of the control perovskite films. For the SEM-HCl-based perovskite films, much stronger diffraction peaks were observed at 14.0° and 28.0°, corresponding to the (100) and (200) crystal planes, however, the peaks at the (220) and (022) crystal planes were lower than those of the control samples. This suggests a relatively ordered arrangement of the tin perovskite grains due to the chemical interactions between the SEM-HCl and FA_0.75_MA_0.25_SnI_3_. The ensuing preferential growth of crystal grains can contribute to reducing the grain boundaries of the perovskite top surfaces, thereby enhancing the charge transport within the SEM-HCl-derived perovskite film.

The surface topography of the corresponding tin perovskite films was also investigated by scanning electron microscopy (Fig. 1b, c and Fig. S1 in Supporting Information). From the SEM images, it can be seen that the grain size of perovskite crystals was reduced, which may have been due to the additives leading to the formation of nucleation sites, thereby optimizing grain boundary defects. Minor pits were discerned on the surface of the control perovskite sample, a feature potentially augmenting nonradiative recombination. The target film based on SEM-HCl showed a noticeable improvement in reducing pits in SEM images. An atomic force microscope (AFM) was also used to further investigate the surface roughness of the corresponding perovskite films in a scanning area of 3.5 × 3.5 μm^2^, as depicted in Fig. 1d, e. Compared to the control film, the root mean square (RMS) roughness value of SEM-HCl-treated perovskite was reduced from 32.3 to 24.1 nm. Thus, the SEM-HCl could contribute to reducing leakage current due to the improved roughness of the films. These characterizations indicated that SEM-HCl could regulate the perovskite crystallization in high-quality films.

The emission properties of the perovskite films deposited on quartz substrates were examined through steady-state photoluminescence (PL) spectroscopy (Fig. 1f). A PL peak was found for the SEM-HCl-derived perovskite films that was intensified in comparison to that of the control sample, thereby indicating that a reduced defect density was created within the FA_0.75_MA_0.25_SnI_3_ sample, based on SEM-HCl additive. In addition, the time-resolved photoluminescence (TRPL) spectra of the control and SEM-HCl-derived samples are shown in Fig. S2, from which charge lifetimes for each spectrum could be fitted (the fitting methods are shown in Supporting Information). The average PL decay lifetime of SEM-HCl-derived perovskite films was remarkably enhanced from 1.29 to 3.91 ns. These results indicate that the charge carrier recombination was also suppressed, matching PL spectra observations.

The UV–vis absorption spectra of the control and SEM-HCl-derived perovskite films were also characterized. As shown in Fig. S3, the SEM-HCl-treated perovskite films exhibited no absorption loss relative to that of the control film, thereby circumventing the current intensity (*J*_sc_) loss typically encountered in additive engineering.

**Fig. 2:**
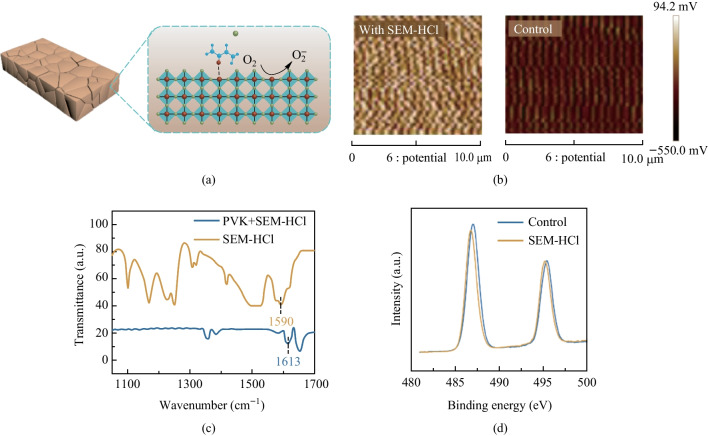
**a** Schematic illustration of the effects of SEM-HCl additive on the tin perovskites. **b** Surface potential distribution of tin perovskite films measured by KPFM. **c** FTIR characterization of C=O peak position shift of SEM-HCl and SEM-HCl + perovskite (PVK) samples. **d** XPS Sn 3d_5/2_ spectra of the corresponding samples

The schematic illustration of the regulation perovskite defects via the SEM-HCl additive is shown in Fig. 2a. To appraise the action process on the tin perovskite surface before and after the introduction of SEM-HCl, electric potential on the surface of the tin perovskite film was analyzed employing a Kelvin probe force microscope (KPFM) (Fig. 2b). After introduction of SEM-HCl additive, a noticeable shift in the contact potential was observed, from approximately − 0.4 eV for the control perovskite film to − 0.1 eV for the SEM-HCl-derived perovskite film, indicating a reduction of hole concentration and Sn vacancies on the surface of target films [38]. The decrease in Sn vacancies in the improved perovskite film indicated a higher concentration of Sn cations, leading to an elevated surface potential. SEM-HClwas shown to significantly diminish concentration of Sn vacancies and self-doping, also reducing hole concentration. This reduction in undercoordinated Sn ions on the perovskite surfaces resulted in a substantial alteration of the contact potential. Therefore, electron cloud density significant increased, contributing to reduction the adsorption of oxygen molecules.

Benefits were conferred by SEM-HCl molecules due to the chemical coordination between Sn^2+^ of perovskite and the O=C–N group. To confirm the corresponding chemical interaction between SEM-HCl and tin perovskite, we utilized a series of characterizations. From the Fourier transform infrared (FTIR) spectroscopy measurements (Fig. 2c), the C=O stretching vibration for the SEM-HCl molecule was located at 1590 cm^−1^, transitioning to 1613 cm^−1^ in the FA_0.75_MA_0.25_SnI_3_ sample with SEM-HCl, demonstrating the interactions between the C=O unit and perovskite. These infrared peak fluctuations showed that SEM-HCl was not merely physically adsorbed onto the perovskite surface to reduce O_2_ molecules absorption but also engaged in chemical interactions with perovskite via the C=O moieties.

X-ray photoelectron spectroscopy (XPS) measurements were also made to further investigate the chemical interactions between SEM-HCl and perovskite (Fig. S4). The Sn XPS spectra in Fig. 2d further confirmed that Sn from FA_0.75_MA_0.25_SnI_3_ was likely the active site of coordination with the O=C group in SEM-HCl, as the peak binding energy of the Sn spectrum displayed a discernible shift, between the control to the SEM-HCl-treated films, toward lower binding energy (~ 0.2 eV). It should be noted that the Sn^4+^ content was markedly diminished in perovskite that contained SEM-HCl additive, signifying that the self-doping effects had been significantly suppressed. Thus SEM-HCl molecule could inhibit the generation of Sn^4+^ and improve the device stability.

**Fig. 3:**
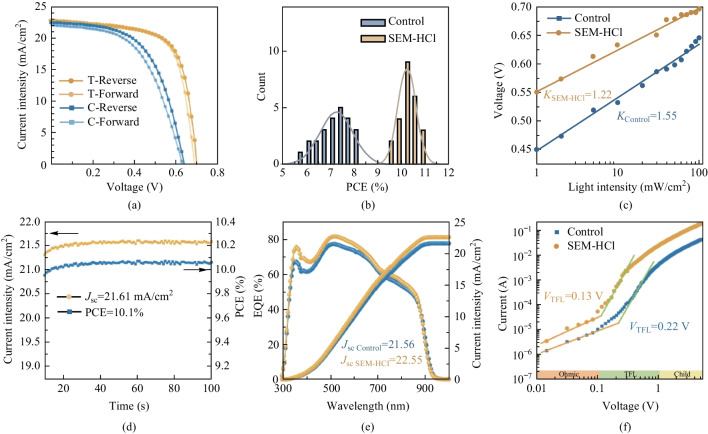
**a** *J–V* curves (reverse and forward scan) of control and SEM-HCl derived devices. **b** Histograms of PCEs measured for 24 devices. **c** Light intensity dependent of *V*_oc_ of TPSCs. **d** Steady-state photocurrent and PCE at a constant bias of 0.5 V for the champion TPSC, **e** EQE spectra of TPSCs. **f** Current–voltage curves from Hole-only devices to calculate the trap-state density

Based on the advantages mentioned above, TPSCs were then fabricated with an ITO/PEDOT:PSS/FA_0.75_MA_0.25_SnI_3_/C_60_/BCP/Al structure, via the introduction of SEM-HCl, as shown in Fig. S5, Supporting Information. Figure 3a and Table S1 display the current density–voltage (*J–V*) and photovoltaic parameters of the freshly prepared control and SEM-HCl-based TPSCs, measured under both forward and reverse scans. Meanwhile, the hysteresis index (HI) was also calculated by the following Eq. (1).

1 $${\text{HI}} = \frac{{{\text{PCE}}_{{\text{R}}} - {\text{PCE}}_{{\text{F}}} }}{{{\text{PCE}}_{{\text{F}}} }} \times 100\% .$$

The corresponding HI values for the control and target TPSCs are 0.078 and 0.018, respectively. Notably, the SEM-HCl-derived TPSCs exhibited a superior PCE and a diminished HI value compared to those of the control device. The optimized TPSC based on SEM-HCl achieved a PCE of 10.9% with a *V*_oc_ of 0.7 V, a *J*_sc_ of 22.80 mA/cm^2^, and a fill factor (FF) of 67.97. This PCE was 32.8% higher than that of the control device (8.2%). To further check *J–V* measurements, the parameter distribution with 24 different devices in one batch were also measured and are shown in Fig. 3b and Fig. S6. It can be seen that the distributions of photovoltaic parameters of target TPSCs were narrower than those of the control devices, indicating superior reproducibility for the SEM-HCl-derived TPSCs. Furthermore, light-intensity-dependent *V*_oc_ curves were created and examined to assess charge recombination losses (Fig. 3c). In the open-circuit condition, *V*_oc_ is determined by the following Eq. (2).

2 $$V_{{{\text{oc}}}} = \frac{{n_{{{\text{id}}}} KT}}{q}\ln \left( {\frac{{J_{{{\text{ph}}}} }}{{J_{0} }} + 1} \right),$$

where *n*_id_ represents the ideality factor, and *J*_ph_ and *J*_0_ are the photocurrent density and saturated current density, respectively. Compared to the control device, the SEM-HCl-derived TPSC exhibited a diminished *n*_id_ from 1.55 to 1.22. This smaller *n*_id_ further indicated reduced charge recombination, leading to enhanced *V*_oc_ and PCE. The electrochemical impedance spectroscopy (EIS) biased at 0.5 V was employed to characterize the interfacial charge transport and recombination processes in the PSCs, with results as shown in Fig. S7. The steady-state current densities and stable output PCE were also scrutinized under continuous sunlight (AM 1.5G) (Fig. 3d). The SEM-HCl-based TPSCs had a stable output PCE and steady-state *J*_sc_ of 10.1% and 21.61 mA/cm^2^, respectively. To validate the reliability of the enhanced *J*_sc_ in the target TPSCs, the external quantum efficiency (EQE) and the corresponding integrated *J*_sc_ curves were also assessed (Fig. 3e). The integrated *J*_sc_ values of the control and SEM-HCl-based TPSCs were 21.56 and 22.55 mA/cm^2^, respectively, which matched well with measured *J*_sc_ values (Fig. 3a). These results substantiated the reliability of our *J–V* measurements.

To further elucidate the effects of the SEM-HCl molecule on the trap-states of perovskite thin films, the space-charge limited current (SCLC) model of hole-only devices was employed (Fig. 3f). The current and voltage exhibited a linear relationship at lower voltages, after which the current escalated exponentially. The voltage at the inflection point is referred to as the trap-state filling limit voltage (*V*_TFL_), which can be ascertained from the *J–V* curve. Equation (3) is typically used to compute the value of trap state density (*n*_trap_) in perovskite films:

3 $$n_{{{\text{trap}}}} = \frac{{2\varepsilon \varepsilon_{0} V_{{{\text{TFL}}}} }}{{qL^{2} }},$$

where *ε*_0_ denotes the vacuum dielectric constant, *ε* represents the relative dielectric constant, *q* is the elementary charge, and *L* is the thickness of the perovskite film. The calculated values of *n*_trap_ for the control and SEM-HCl-derived perovskite films were 3.52 × 10^16^ and 2.08 × 10^16^ cm^−3^, respectively, indicating the SEM-HCl additive could significantly reduce the trap-states of the tin perovskite film. This was in line with the enhanced efficiency of the SEM-HCl-treated PSCs.

**Fig. 4:**
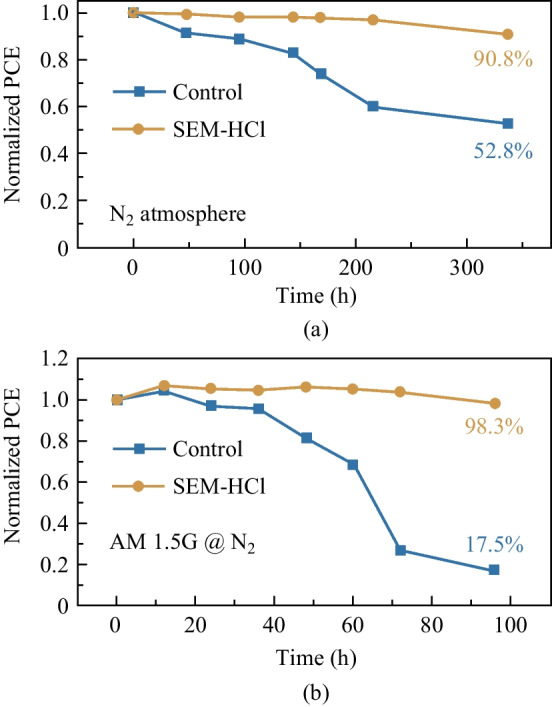
Device stability, **a** without encapsulation in glove box, **b** and under continuous AM 1.5 light illumination

Stability is still a substantial impediment in TPSCs further development; we further examined the corresponding stability of the control and target devices under different conditions (glove box and in N_2_ under continuous light); the results are as shown in Fig. 4. The initial PCEs of target and control device under N_2_ condition were 10.85% and 8.17%, respectively. It was observed that the stability of the SEM-HCl-treated device barely decreased in glove box conditions for more than 300 h. Also, the SEM-HCl-treated TPSC showed higher illumination stability. The initial PCE of the target device under continuous illumination was 10.43%. After exposure to continuous AM1.5 illumination for 100 h, the SEM-HCl-derived device showed almost no decline in PCE, while that of the control device was less than 20% of the initial value after 100 h. The enhanced illumination stability may have been due to the light soaking effect and reduction of defect concentration by the additive.

## Conclusion

In summary, high-quality tin perovskite films with low deep-level trap state densities were fabricated by introducing SEM-HCl into the perovskite precursor. The N–C=O functional group in SEM-HCl were bound to the uncoordinated Sn^2+^ of the tin perovskites, thus reducing the deep-level trap states though suppressing the adsorbed oxygen molecules. In addition, the existence of SEM-HCl also significantly improved the morphology and crystallization of the resulting tin perovskite films, effectively curbing the oxidation of Sn^2+^. Consequently, the TPSCs based on 3 mol% SEM-HCl additive achieved a peak PCE of 10.9%, while also demonstrating improved stability in glove box and AM1.5 illumination conditions. This work could provide a possible direction for inhibition of oxygen molecule adsorption on the surface of tin perovskites in the presence of light, and the suppression of oxygen molecule = reactivity with photogenerated charges resulting in formation of superoxide molecules. Thus the work provides a guideline for construction of high-performance TPSCs.

### Supplementary Information

Below is the link to the electronic supplementary material.

Supplementary file1 (PDF 786 KB)

## Data Availability

The data that support the findings of this study are available from the corresponding author, upon reasonable request.
