# Supplementary material for: Suppression of deep-level traps via semicarbazide hydrochloride additives for high-performance tin-based perovskite solar cells
Source: Front Optoelectron. 2023 Dec 29;16(1):47. doi: 10.1007/s12200-023-00103-1 (PMC10754768; doi:10.1007/s12200-023-00103-1)
Supplement: Supplementary file 1 — Supplementary file1 (PDF 786 KB) [file 12200_2023_103_MOESM1_ESM.pdf]

Supporting Information

Deep-level traps suppressed via semicarbazide hydrochloride additives for high-performance tin-based perovskite solar cells.

Wenbo Jia[1], Yi Jing[1], Han Zhang[1], Baoyan Tian[2], Huabo Huang[2], Changlei Wang[3], Ligang Xu[1,4]**

[1]Key Laboratory for Organic Electronics and Information Displays (KLOEID) & Jiangsu Key Laboratory for Biosensors, Institute of Advanced Materials (IAM), Nanjing University of Posts & Telecommunications, 9 Wenyuan Road, Nanjing, China.

[2] Hubei Key Laboratory of Plasma Chemistry and Advanced Materials, Key Laboratory for Green Chemical Process of Ministry of Education, School of Materials Science and Engineering, Wuhan Institute of Technology, Wuhan 430205, China.

[3]School of Optoelectronic Science and Engineering & Collaborative Innovation Center of Suzhou Nano Science and Technology; Key Lab of Advanced Optical Manufacturing Technologies of Jiangsu Province & Key Lab of Modern Optical [4]Technologies of Education Ministry of China, Soochow University, Suzhou, China. Wuhan National Laboratory for Optoelectronics, Huazhong University of Science and Technology, 1037 Geyu Road, Wuhan, Hubei, China.

E-mail: hbhuang@wit.edu.cn; iamlgxu@njupt.edu.cn

Content

1. Materials

2. Device fabrication

3. Characterization

4. Figure parts

1. Materials.

Poly(3,4-ethylenedioxythiophene):poly(styrenesulfonate) (PEDOT:PSS) water dispersion was obtained from Heraeus (Clevios P VP.Al 4083). Formamidinium iodide (FAI) (≥ 99.5%) and methylammonium iodide (MAI) (≥ 99.5%) was purchased from Xi'an Polymer Light Technology Co., Ltd. Tin iodide (SnI_2, 99.99%) and tin fluoride (SnF_2, 99%) were from Sigma-Aldrich Co., Ltd and Aladdin, respectively. Semicarbazide hydrochloride (SEM-HCl) (≥ 98%) was purchased from Energy Chemical Co., Ltd. Fullerene (C_{60}) and bathocuproine (BCP, 99.7%) were obtained from Luminescence Technology Co., Ltd. Solvents of N, N-dimethylformamide (DMF, 99.5%), dimethyl sulfoxide (DMSO, > 99.9%) and chlorobenzene (CB, 99.5%) were from either Acros Organics, Alfa Aesar, or TCL (Shanghai) Development Co., Ltd. MoO_3 and Al were purchased from Ji'lin OLED Co., Ltd and Zhong Nuo Advanced Material (Beijing) Technology Co., Ltd, respectively. All these materials were used as received without further treatment.

2. Device fabrication.

Indium tin oxide (ITO) deposited glass substrate was cleaned in deionized (DI) water, acetone, and ethanol subsequently and respectively for 20 min in an ultrasonicator (Shumei KQ300DE). After dried with nitrogen gas, the ITO surface was treated with UV-ozone using an ODT UV-O3 Cleaner for 15 min. Then, PEDOT:PSS was spin-coated on ITO at 4000 rpm for 60 s in air, followed by annealing at 120°C for 20 min. The PEDOT:PSS-coated ITO was then transferred into a glove box filled with high-purity N_2. The Sn perovskite precursor was prepared by mixing FAI, MAI, SnI_2

and SnF_2 at a molar ratio of 0.75:0.25:1:0.1 with a concentration of 1.0 mol/L in the mixed solvent of DMF/DMSO (4/1, v/v) and stirring at 25°C for 2 h. For the SEM-HCl-derived precursor solution, the molar ratio of SEM-HCl and SnI_2 was 3%. Then perovskite precursor was spin-coated on the surface of ITO/PEDOT: PSS substrates at 5000 rpm for 30 s, then 150 μL of chlorobenzene was quickly dropped on the center of the substrates at the 13th s of the spin-coating process to produce the dense perovskite crystal film followed by 70°C thermal annealing for 10 min. Finally, the C_{60} (20 nm), BCP (6 nm) and Al (100 nm) layers were sequentially evaporated on the surface of perovskite films. The thickness was precisely controlled by crystal oscillator during the vapor deposition process. The area of the Al electrode was determined by a mask and the active area of a single device is 0.09 cm^2.

3. Characterization

The fabricated devices were measured without encapsulation at room temperature (25°C) in N_2 atmosphere. Their photocurrent density-voltage (*J–V*) curves and power conversion efficiencies (PCEs) were obtained by a computer-programmed Keithley 2400 source/meter under 100 mW/cm^2 illumination of AM 1.5G solar simulator (SAN-EI Electric Co., Ltd.). The external quantum efficiency (EQE) measurements were conducted in air by QE-R system (Enli Technology Co., Ltd.). The SCLC characterization was performed on hole-only devices with the structure of ITO/PEDOT: PSS (~ 40 nm)/perovskite (130~160 nm)/MoO_3 (10 nm)/Al (100 nm), all devices were measured from 0 to 7 V with a step size of 0.02 V under dark conditions using the *J-V*

sweep mode developed by a Keithley 2400 source/meter unit.

UV-vis absorption spectra were obtained on a Jasco V-750 UV-Visible/Near-Infrared Spectrophotometers. The Fourier Infrared spectra were characterized by PE-Spectrum Two in transmittance model. X−ray diffraction (XRD) and scanning electron microscopy (SEM) characterizations of $FA_{0.75}MA_{0.25}SnI_3$ films were conducted on Rigaku Smart lab X-Ray diffractometer and Hitachi S-4800 scan electron microscope, respectively. Steady-state photoluminescence (PL) spectra were collected using Hitachi F-4600 spectrofluorometer (Japan). The time-resolved PL decays of the perovskite films were measured by an Edinburgh FLS 980. A 450 nm pulse laser with a repetition frequency of ~1–20 MHz was employed to measure the PL lifetime by a fitted bi-exponential decay model ($I=I_1\times\exp[-(t/\tau_1)]+I_2\times\exp[-(t/\tau_2)]$). X-ray photoelectron spectroscopy (XPS) experiments were carried out on an ESCALAB 250 system equipped with a monochromatic Al Kα X-ray source (hv=1486.6 eV). The atom force microscopy (AFM) measurements were performed at room temperature using Bruker Dimension Icon AFM equipped with Scanasyst-Air peak force tapping mode AFM tips from Bruker. The electrochemical impedance spectra (EIS) were measured on a CHI660 electrochemical workstation (CH Instrument Inc.). A 20 mV voltage perturbation was applied at different direct current voltages ranging from 0 to 0.5 V with frequencies between 105 and 1 Hz under dark conditions. The results were fitted using the software of Zsim. All characterizations and measurements were performed in ambient conditions.

4. Figure parts

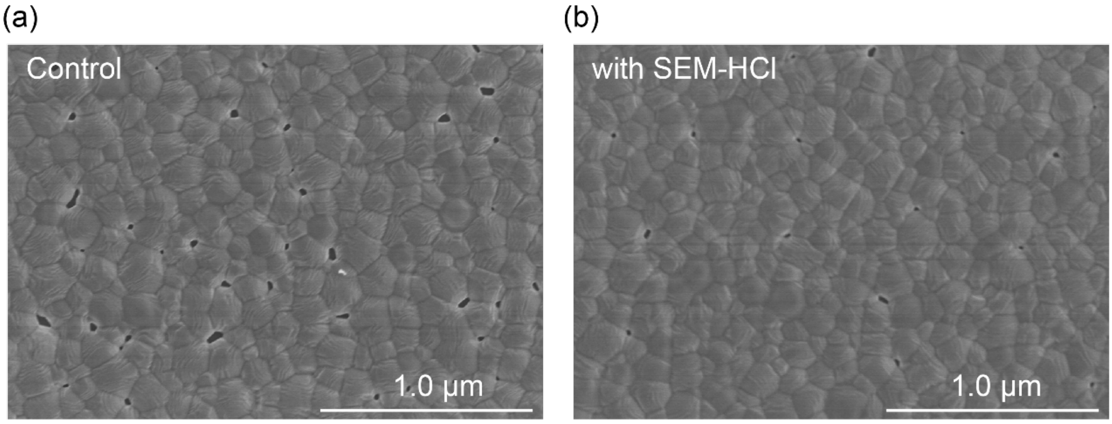

Fig. S1. Top-view SEM images of the perovskite films at low magnifications.

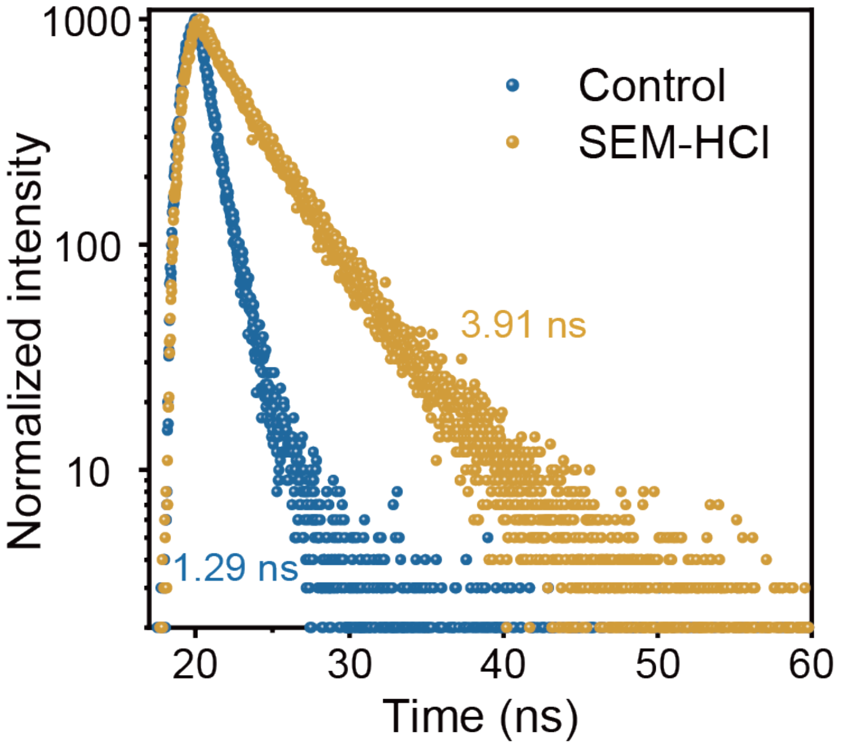

Fig. S2. Time-resolved PL decay profiles of the control and SEM-HCl-derived perovskite films.

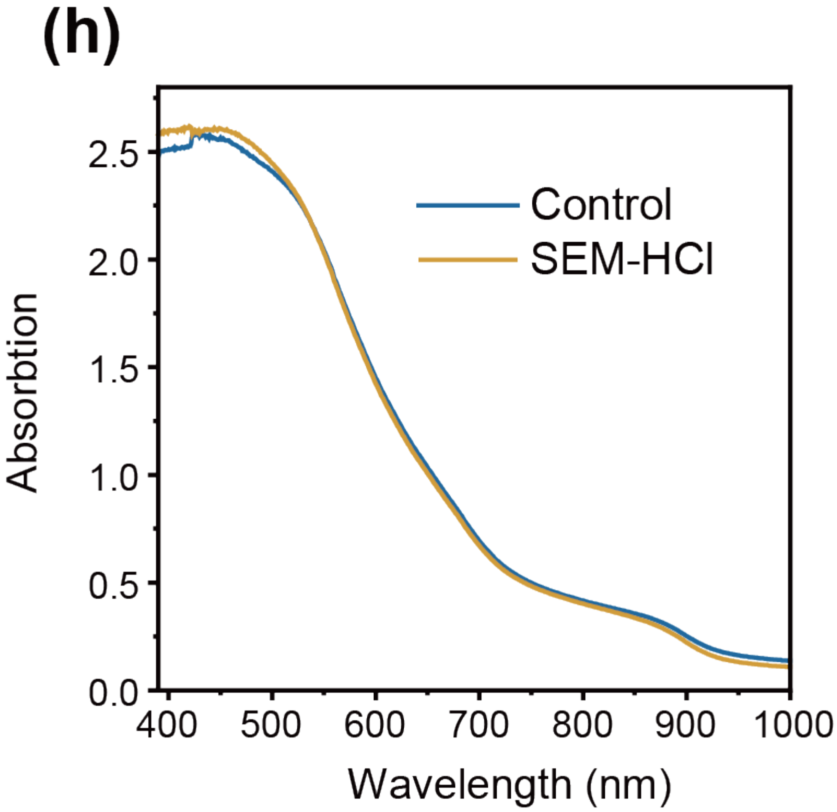

Fig. S3. Absorption spectra of the control and SEM-HCl-derived perovskite films.

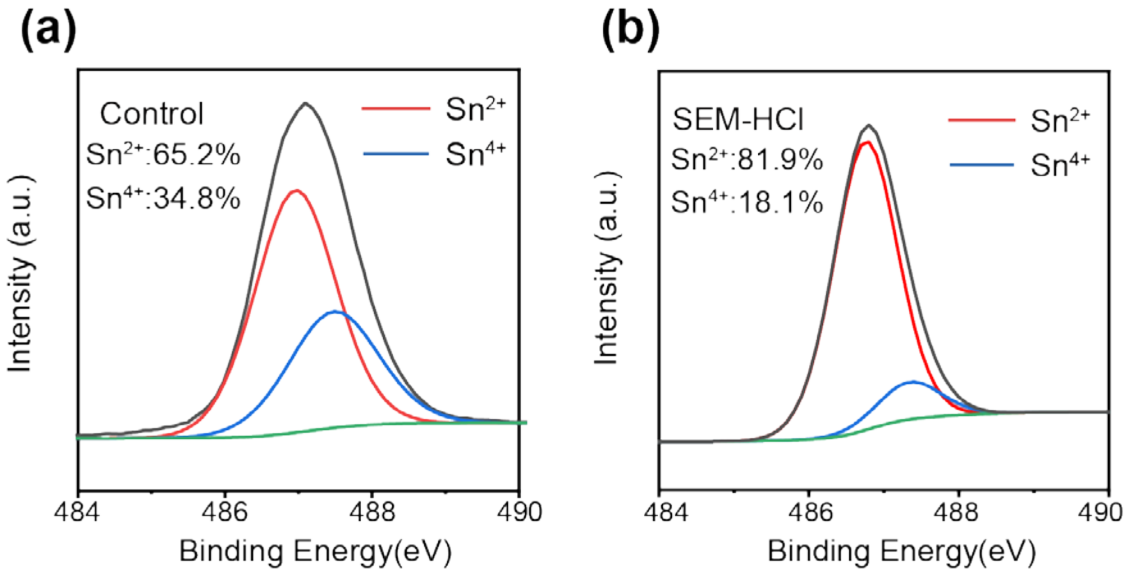

Fig. S4. XPS Sn $3d_{5/2}$ spectra of the control (a) and SEM-HCl-derived perovskite films (b).

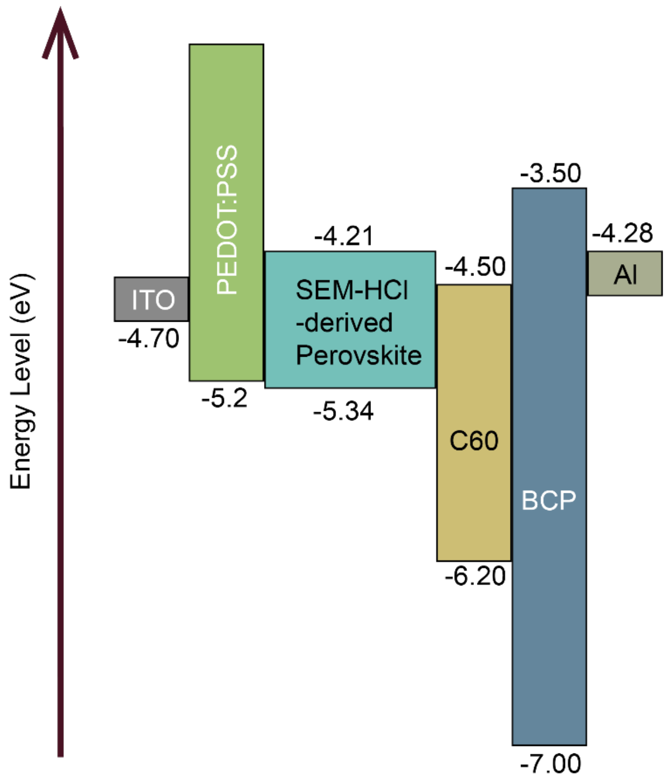

Figure S5. Energy Level diagram of the SEM-HCl derived TPSCs.

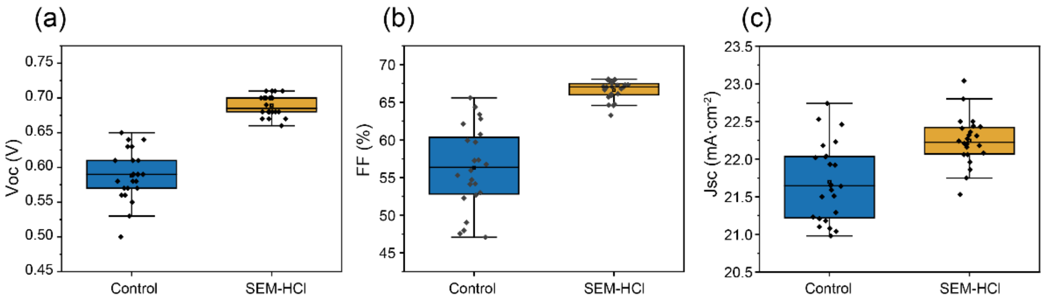

Figure S6. Device performance statistics on (a) V_{oc}, (b) FF and (c) J_{sc} of the control and SEM-HCl derived TPSCs.

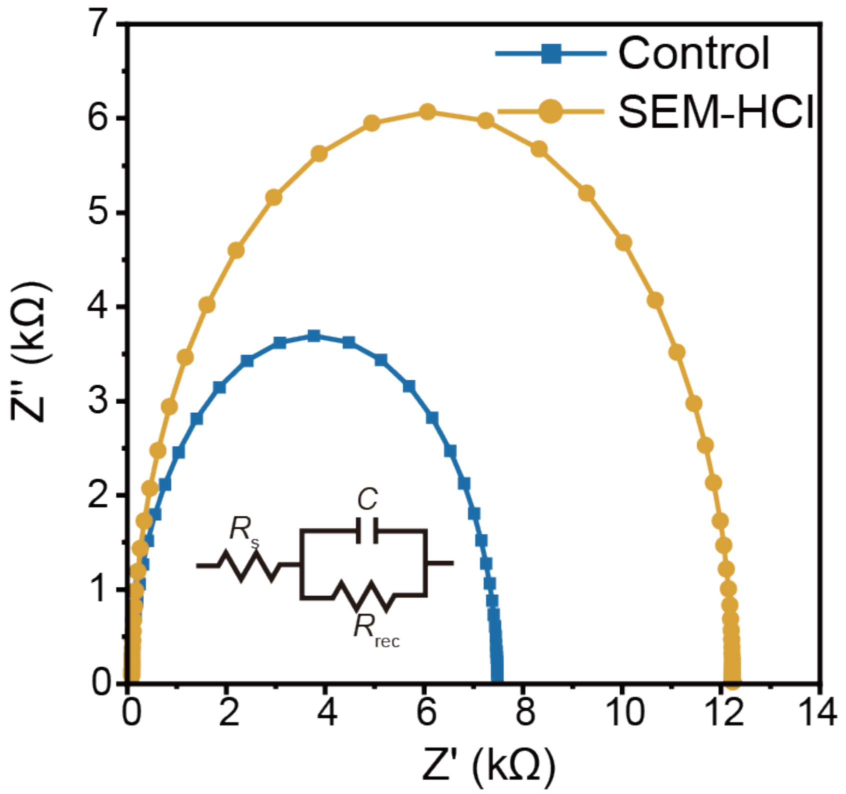

Fig. S7. Nyquist plots of the EIS measurements of control and SEM-HCl-derived TPSCs under dark condition.

Table S1. Photovoltaic parameters of the control (w/o) and SEM-HCl-derived TPSCs (3 mol% SEM-HCl) based on Forward and Reverse scans.

	V_{oc} (V)	J_{sc} (mA cm^{-2})	FF (%)	PCE (%)
w/o-Reverse	0.64	22.46	56.76	8.17
w/o-Forward	0.63	22.04	54.11	7.53
3 mol%-Reverse	0.70	22.80	67.97	10.85
3 mol%-Forward	0.69	22.53	68.58	10.65
